# Breastfeeding Continuation at One Month Postpartum Among Women in Treatment for Opioid Use Disorder Who Initiated Breastfeeding: Prevalence and Determinants

**DOI:** 10.1177/26884844251364705

**Published:** 2025-08-01

**Authors:** Julia Eisenberg, Meghan Gannon, Kim McLaughlin, Diane J. Abatemarco, Vanessa L. Short

**Affiliations:** ^1^ Sydney Kimmel Medical College, Thomas Jefferson University, Abington, Pennsylvania, USA.; ^2^ College of Nursing, Thomas Jefferson University, Phila, Pennsylvania, USA.

**Keywords:** breastfeeding, breastfeeding determinants, breastfeeding duration, opioid use disorder, postpartum

## Abstract

**Objective:**

To describe breastfeeding behaviors and determinants in the 1-month postdelivery period among women in treatment for opioid use disorder.

**Study Design:**

Participants completed one questionnaire during pregnancy and one questionnaire at 1 month postpartum. Those who reported on the postpartum questionnaire that they had initiated breastfeeding were included in this analysis (*N* = 31). Infant feeding practices, receipt of lactation support, and demographic, psychosocial, and infant birth characteristics were compared between those who breastfed for at least 1 month and those who did not.

**Results:**

In all, 45% of the participants were breastfeeding at 1 month post delivery. Among those who discontinued breastfeeding by 1 month post delivery, two-thirds reported that they had not breastfed for as long as they wanted. Breastfeeding continuation at 1 month was more common in participants who expressed prenatal breastfeeding intention, had shorter infant hospital length of stays, received lactation materials/support, and reported lower stress and depressive symptoms. Among those who stopped breastfeeding at 1 month, perception of low breast milk supply was the most frequently cited reason.

**Conclusions:**

Lactation support programs are needed to help women with opioid use disorder meet their infant feeding goals. Such programs may want to consider simultaneously addressing maternal psychosocial factors.

## Introduction

Several leading national health organizations, including the American College of Obstetrics and Gynecologists,^1^ the American Academy of Pediatrics,^2^ and the Academy of Breastfeeding Medicine,^3^ support breastfeeding in women with opioid use disorder (OUD) who are on methadone or buprenorphine unless specifically contraindicated. As initiation rates of breastfeeding for women with OUD have been increasing throughout recent decades,^4–7^ recent research suggests that women with OUD now breastfeed at similar rates compared with the general population of recently delivered women in the United States. The duration of breastfeeding among this population, however, remains significantly shorter.^4^ Because breastfeeding has numerous maternal and infant benefits, increasing breastfeeding duration among women in treatment for OUD could have important clinical and public health implications.

Maternal benefits of breastfeeding include greater birth spacing, longer periods of amenorrhea, and a reduction of breast cancer, type 2 diabetes, and hypertension risk.^8,9^ Women with OUD have articulated breastfeeding as a source of maternal−infant bonding.^10^ In addition to providing nutrients to the child, breast milk positively influences neurodevelopment and offers protections against neonatal morbidity and mortality.^11,12^ There is also evidence that breastfeeding has unique benefits for infants exposed to opioids *in utero*. Studies have shown that breastfed infants treated for neonatal opioid withdrawal syndrome (NOWS)—a withdrawal syndrome that can occur in newborns exposed to certain substances, including opioids, *in utero*—have delayed onset of NOWS, reduced NOWS severity, and shorter hospital stays compared with infants who are not breastfed.^13–15^ While several studies have examined breastfeeding continuation at hospital discharge among women with OUD,^6^ little is known about breastfeeding behaviors beyond the birth hospitalization, and few studies have described non-hospital-related reasons for breastfeeding discontinuation in this population. The objectives of this study were to describe infant feeding practices during the 1-month postpartum period among a cohort of women receiving pharmacotherapy for OUD who had initiated breastfeeding and to examine both hospital- and non-hospital-associated factors associated with breastfeeding at 1 month postpartum. Identifying factors that influence infant feeding practices could help design programs that aim to increase breastfeeding rates, which would ultimately positively impact the mother–infant dyad.

## Materials and Methods

### Study design

This was an analysis of data collected from a longitudinal study to describe determinants of breastfeeding intention, initiation, and duration among women in treatment for OUD. Pregnant women who were receiving treatment, including pharmacotherapy plus counseling, for OUD from a single, urban opioid treatment program were recruited in their third trimester between the years of 2019 and 2021. Inclusion criteria included: (1) pregnant and at least 29 weeks’ gestation, (2) ability to read English, and (3) plans to parent infant after delivery. Individuals were excluded if they did not have plans to parent infant after delivery. All materials and procedures for this study were approved by the Jefferson Institutional Review Board.

### Measures

For this study, data from questionnaires self-administered during the third trimester and at 1 month postpartum were utilized. No identifiable information was recorded on the questionnaires and, when possible, validated survey items from the Pregnancy Risk Assessment Monitoring System (PRAMS)^16^ were used. Additionally, data were abstracted from the mother’s birth hospital electronic medical record (EMR).

#### Demographics

On the prenatal questionnaire, participants were asked to report the following: age, race, ethnicity, relationship status, highest level of education, pregnancy intention, breastfeeding intention, current tobacco use, number of children, and history of breastfeeding.

#### Lactation support

On the 1-month postpartum survey, participants were asked if during pregnancy: (1) someone answered breastfeeding-related questions (yes, no), (2) they were offered a breastfeeding class (yes, no), and (3) they attended a breastfeeding class (yes, no). Participants were additionally asked to identify support received during the birth hospital stay by selecting from a list of possible supports. Finally, participants were asked about various lactation supports in the first month postpartum.

#### Breastfeeding initiation and continuation

Breastfeeding initiation was measured with the validated PRAMS question^17^ on the 1-month postpartum survey: “Did you ever breastfeed this baby (or feed this baby your pumped milk)?” If yes, “Are you currently breastfeeding or feeding pumped milk to your new baby?” For those who responded that they were no longer breastfeeding, two follow-up questions were asked: “How old was your baby when you completely stopped breastfeeding or feeding pumped milk to your new baby?” and “Did you breastfeed for as long as you wanted to?” They were also asked to select from a list of reasons for early cessation of breastfeeding.

#### Birth characteristics

The following infant birth characteristics were abstracted from the mother’s EMR: delivery mode, gestational age at birth, birthweight, NOWS treatment, neonatal intensive care unit (NICU) admission, and hospital length of stay (LOS).

#### Postpartum psychosocial factors

The Edinburgh Postnatal Depression Scale (EPDS) score abstracted from the mother’s EMR was used to evaluate depression in the early postpartum period. Two PRAMS questions were asked on the 1-month postpartum survey to further assess symptoms of depression. Specifically, participants were asked whether they had felt down since their baby was born (yes, no) and if they had little interest since the baby was born (yes, no). Finally, the 10-item Perceived Stress Scale (PSS)^18^ was included on the 1-month postpartum survey and used to measure general stress. Participants were asked to rate 10 items using a five-point Likert scale ranging from “never” to “very often” and subsequently received a total stress score between 0 and 40, with higher scores representing higher perceived stress.

### Statistical analyses

Data were described using descriptive statistics. Frequency counts and percentages and medians and ranges were used to describe data. Due to the small sample size, we did not compare data between the two groups. All statistical analyses were performed using SAS version 9.4 (SAS Institute, Cary, NC, USA).

## Results

Of the 65 participants who completed the prenatal questionnaire, 42 (65%) completed the 1-month postpartum questionnaire. Participants who reported that they had initiated breastfeeding (*n* = 31) on the 1-month postpartum questionnaire were included in this analysis. Those who did not complete the 1-month postpartum questionnaire were considered lost to follow-up. There were no differences in demographic characteristics between those lost to follow-up and those who completed the 1-month postpartum questionnaire.

Most participants were White (71%), non-Hispanic (84%), and married or in a committed relationship (58%) and had no more than a high school education (61%). Most (87%) reported prenatal intention to breastfeed, while less than one-third (29%) reported on the prenatal survey that they were ready to be pregnant. Most participants (81%) had at least one additional child. Among those who had other children, more than half (52%) reported a history of breastfeeding.

**FIG. 1.**
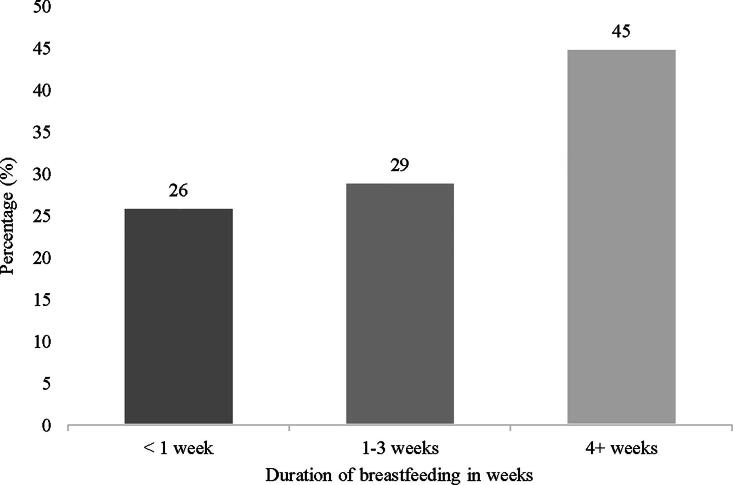
Duration of breastfeeding among those who reported breastfeeding initiation.

Among those who reported that they intended to breastfeed on the prenatal survey, the median length of intended breastfeeding duration was 12 months (range: 1–24 months). Despite this, more than half (55%) of the participants stopped breastfeeding by the 1-month postpartum visit. Figure 1 shows the breastfeeding duration of the participants. At 1 month postpartum, 45% of those who had initiated breastfeeding were still breastfeeding. Among those who had discontinued breastfeeding by 1 month postpartum, two-thirds (65%) reported that they did not breastfeed for as long as they wanted. The most frequently selected reasons for discontinuing breastfeeding earlier than planned included: “I did not have enough milk” (63%), “My baby was in the NICU” (62%), “My baby had trouble sucking or latching on” (62%), “My methadone treatment made it too difficult” (50%), “My baby got sick” (43%), “Breast milk alone did not satisfy my baby” (40%), and “It was too painful or too difficult” (37%).

For the most part, maternal demographic and health characteristics seemed to be similar between the women who continued breastfeeding at 1 month and women who did not (Table 1). All women who were still breastfeeding reported prenatal intention to breastfeed compared with 76% of women who had discontinued breastfeeding. The median infant LOS was shorter for those who continued breastfeeding compared with those who did not (11 days vs. 21 days).

**Table 1.** Maternal and Infant Characteristics by 1-Month Postpartum Breastfeeding Status Among Women in Treatment for Opioid Use Disorder Who Reported Breastfeeding Initiation

	Breastfeeding at 1 month *n* = 14 %	No breastfeeding at 1 month *n* = 17 %
Maternal age (in years), median (range)	28.5 (22, 35)	29 (21, 33)
Race		
Non-White	21	35
White	79	65
Ethnicity		
Hispanic	14	18
Non-Hispanic	86	82
Marital status		
Married or in a committed relationship	50	65
Not married or not in a committed relationship	50	35
Parity		
Nulliparous	7	29
Multiparous	93	71
Highest level of education		
Less than high school	14	41
High school	36	29
More than high school	50	29
Ready to be pregnant		
Yes	36	24
No or not sure	64	76
History of breastfeeding		
Yes	69	33
Prenatal breastfeeding intention		
Any intent to breastfeed	100	76
No intent to breastfeed	0	24
Mode of delivery		
C-section	50	33
Vaginal	50	67
NICU admission		
Yes	79	93
Infant treated pharmacologically for NOWS		
Yes	57	73
Low infant birthweight		
Yes (<2500 g)	14	41
Infant LOS (in days), median (range)	11 (142)	21 (141)
Infant gestational age at birth (in weeks), median (range)	38 (32, 40)	37 (31, 39)

LOS, length of stay; NICU, neonatal intensive care unit; NOWS, neonatal opioid withdrawal syndrome.

As shown in Table 2, women who were still breastfeeding at 1 month postpartum were more likely to report that during the birth hospital stay, hospital staff gave them information about breastfeeding (100% vs. 64%) and a telephone number to call for help with breastfeeding (71% vs. 24%) compared with those who were not breastfeeding at 1 month postpartum. Scores on the PSS varied between the two groups; scores were greater among women who discontinued breastfeeding compared with those who continued breastfeeding, indicating greater general stress among those who ceased breastfeeding. Nearly four times as many women who discontinued breastfeeding reported on the 1-month postpartum survey that they had experienced little interest since the baby was born compared with women who continued breastfeeding (53% vs. 14%).

**Table 2.** Prenatal and Postpartum Supports and Psychosocial Factors Associated with Breastfeeding Continuation at 1 Month Postpartum Among Women Who Initiated Breastfeeding

	Continued breastfeeding at 1 month *n* = 14 %	Did not continue breastfeeding at 1 month *n* = 17 %
Prenatal support		
Someone answered breastfeeding-related questions	57	65
Offered breastfeeding class	43	18
Attended breastfeeding class	21	12
Hospital-based postpartum support		
Hospital staff gave me information about breastfeeding	100	64
Hospital gave a breast pump	86	82
Baby stayed in the same room with me at the hospital	86	53
Hospital staff physically helped me learn how to breastfeed	57	53
Hospital gave a telephone number to call for help with breastfeeding	71	24
I breastfed in the first hour after my baby was born	64	35
My baby was fed only breast milk at the hospital	21	0
Non-hospital-based postpartum support		
A home visiting nurse talked to me about breastfeeding^a^	62	30
Received breastfeeding information from WIC^a^	40	35
Someone close to me (family, friend) suggested I should not breastfeed	21	36
Health care worker talked with me about breastfeeding	57	41
Postpartum psychosocial factors		
Feeling down since the baby was born	31	53
Little interest since the baby was born	14	53
PSS score, median (IQR)	9.5 (2, 29)	18 (6, 27)
EPDS score, median (IQR)	4 (0, 16)	8.5 (0, 18)

^a^ Among those receiving the service.

EPDS, Edinburgh Postnatal Depression Scale; IQR, interquartile range; PSS, Perceived Stress Scale; WIC, Women, Infants and Children Program.

## Discussion

Our findings indicate that among a sample of mothers in treatment for OUD who initiated breastfeeding, duration of breastfeeding was short, and the majority did not breastfeed for as long as they had planned. Additionally, breastfeeding duration was longer among those who expressed prenatal intention to breastfeed, had shorter infant hospital LOS, received lactation materials/support, and had better postpartum psychosocial outcomes (lower perceived stress and/or depressive symptoms). Participants with higher scores on validated assessments, including the EPDS and the PSS, were less likely to report continuation of breastfeeding at the 1-month follow-up visit. However, due to the small sample sizes, data were not compared with statistical tests between those who continued breastfeeding for 1 month and those who did not.

The mean duration of breastfeeding among our study sample, 21 days, aligns with a previous study that reported mean duration of breastfeeding of approximately 3 weeks among women receiving medication for OUD.^19^ One possible explanation for short breastfeeding duration in our sample could be related to OUD treatment itself. At the clinic where this study occurred, most patients receive methadone, which often necessitates traveling to the treatment facility frequently for pharmacotherapy dispensing, counseling, and other components of treatment.^19^ Indeed, nearly half of the mothers in our study indicated that their being in OUD treatment influenced their infant feeding behaviors. Integrating breastfeeding support into OUD treatment programs may be one way to help women meet their breastfeeding goals by providing education, physical lactation support, and space for pumping breast milk. Furthermore, taking into consideration low prenatal health care utilization rates of this population^20,21^ coupled with this study’s high rate of intention to breastfeed but low rate of preparedness for pregnancy, the OUD treatment setting may be a suitable location for prenatal health education to improve breastfeeding initiation and duration.

Hospital-based factors of breastfeeding discontinuation include longer infant hospitalizations, which is consistent with prior research demonstrating longer infant LOS as a barrier to breastfeeding among women with OUD.^6^ Previous studies have also demonstrated an inverse relationship between breastfeeding and LOS among infants exposed to opioids *in utero*.^13,14^ It is possible that infants with longer hospitalizations experienced greater morbidity and/or were at higher risk of feeding issues (*e.g.,* latching), which made breastfeeding continuation difficult. Long hospital stays may also prevent physical connections between mother–infant dyads. Rooming-in, where infants remain with their mothers or family members during their entire hospital stay, was linked to significantly better clinical outcomes compared with traditional care provided in neonatal intensive care units^22^ and may allow for more opportunities for breastfeeding among women with OUD. Exploring additional ways to mitigate the potential negative impact that a long hospital stay has on breastfeeding would be worthwhile.

Our results are similar to other studies that have reported hospital-based predictors of breastfeeding,^23^ including health care provider support and counseling about breastfeeding,^24–27^ as well as birthing facility maternity practices.^28^ Among women with OUD, barriers to breastfeeding in the hospital setting include a lack of support and education from health care providers,^29^ while encouragement from providers^19^ and rooming-in^22^ have been reported to support breastfeeding. Two of the hospital-based supports that we assessed—receiving information about breastfeeding and receiving contact information for lactation support—were more commonly reported by women who continued breastfeeding. This suggests that informational-based supports benefit women who are interested in breastfeeding. While there is some evidence that targeted breastfeeding educational programs seem to be effective at increasing breastfeeding initiation rates in women with OUD,^30^ additional research is needed to examine the specific information and messages that most benefit this patient population. The existing literature does suggest, though, that women with OUD could benefit from education on the benefits and safety of breastfeeding with OUD pharmacotherapy.^31^ Any program that encourages a mother with OUD to breastfeed does need to consider the presence of contraindications, including a woman’s abstinence from illicit drug use and engagement in both prenatal care and substance use treatment.^3^

Study data also suggest that other nonhospital factors influence breastfeeding continuation. Women in our study reported concerns about low milk production, which has been previously reported among the general population and is not unique to this population.^19^ However, NOWS can impact the motor functioning of the infant and affect his or her ability to latch to the breast, which can subsequently cause decreased milk transfer and contribute to decreased milk supply of the mother.^19,32^ As this can be discouraging for the mother, consultation with a lactation specialist is recommended to help improve nursing techniques. Our participants also reported health and OUD treatment-related factors as reasons for discontinuing breastfeeding, including fear of medication transmission through breast milk. This suggests that women receiving pharmacotherapy for OUD may require additional education and assurance regarding medication transmission through breast milk. Additionally, self-reported depression symptoms and stress were greater in those who did not continue breastfeeding, suggesting that recently delivered mothers may need enhanced support to address mental health so it does not interfere with infant feeding. Comorbid psychiatric disorder and OUD are not uncommon,^33^ and pregnancy and the peripartum period have been associated with increased rates of anxiety and depression in pregnant and parenting women with OUD.^34^ However, little is known about the prevalence of psychiatric conditions on women with OUD and on its association with breastfeeding initiation and continuation. The impact that comorbid psychiatric conditions during and after pregnancy has on breastfeeding among women with OUD deserves further study.

The longitudinal nature of this study and collection of patient-reported data beyond hospital discharge are major strengths of this study. Previous studies of breastfeeding among women with OUD have been limited in that they were cross-sectional or retrospective in nature, assessed only unmodifiable clinical or sociodemographic characteristics, included only women who intended to breastfeed, or did not provide information on infant feeding practices beyond hospital discharge. Still, despite the strengths of the current study, potential limitations are noted. The small sample size limited our ability to test for statistically significant differences between the two study groups. Recruitment from one urban opioid treatment program may limit the generalizability of our findings. Recall bias may have been present given that the study relied on the respondent’s self-report. Data are cross-sectional, and we cannot conclude causality between receipt of lactation messages/support and breastfeeding. We did not assess other factors that may influence infant feeding decisions and are prevalent among women with OUD, such as trauma, stigma, and discrimination. Due to the COVID-19 pandemic, in-person research was suspended at the study site during the active data collection period, which impacted our ability to recruit a larger sample size.

In summary, this study indicates that among women in treatment for OUD who initiated breastfeeding, more than half discontinued breastfeeding within 1 month of delivering. Because most study participants reported that they had not breastfed for as long as they wanted to, support programs are needed to help women meet their infant feeding goals. Such programs may want to consider simultaneously addressing maternal psychosocial health, including depression, and incorporating stress reduction techniques. Additionally, testing innovative approaches to lactation education and support, such as integrating programs into opioid treatment settings, may be worth exploring.

## Abbreviations Used

EMR: electronic medical record
EPDS: Edinburgh Postnatal Depression Scale
IQR: interquartile range
LOS: length of stay
NICHD: National Institute of Child Health and Human Development
NICU: neonatal intensive care unit
NOWS: neonatal opioid withdrawal syndrome
OUD: opioid use disorder
PSS: Perceived Stress Scale
PRAMS: Pregnancy Risk Assessment Monitoring System
WIC: Women, Infants and Children Program
